# Beyond the heart: multisystem complications fuelling cardiac dysfunction in myotonic dystrophy type 1

**DOI:** 10.1093/ehjopen/oeae096

**Published:** 2024-11-13

**Authors:** Sofia Khaja

**Affiliations:** CUNY School of Medicine, Townsend Harris Hall, 1589 Amsterdam Ave, New York, NY 10031, USA

**This correspondence refers to ‘CTG repeat length underlying cardiac events and sudden death in myotonic dystrophy type 1’, by H. Itoh**  ***et al*****., https://doi.org/10.1093/ehjopen/oeae078.**

With great interest, we read the paper by Itoh *et al*.^1^ discussing the emerging associations between CTGn repeat expansion in myotonic dystrophy type 1 (DM1) and cardiovascular participation. This study showed that longer CTGn is at a higher risk for cardiac abnormalities and mortality in patients with DM1. Although this study touches upon other causes of death, such as respiratory-related deaths and nutritional support, further investigating non-cardiac outcomes and external causes of death would increase our understanding of how systemic issues interact with cardiovascular health.

Firstly, the intricate relationship between the pulmonary circulation and cardiovascular systems in DM1 is a complex issue. Respiratory insufficiency due to weakened muscles in DM1 may increase the risk of arrhythmias and potentially lead to heart failure.^2^ The study mentions that respiratory-related deaths were highest among patients with the longest CTG repeats, affecting overall mortality.^1^ Therefore, further examining respiratory complications that can worsen cardiac health can clarify the nuanced causes of mortality. Many patients with myotonic dystrophy could also develop sleep apnoea due to weakened respiratory muscles, leading to hypoxic conditions that could increase myocardial oxygen requirement, further increasing the risk of heart failure and hypertension.^2^ This correlation between pulmonary insufficiencies and cardiac health further provides insight into the multisystemic implications of myotonic dystrophy.

In addition, this study indicates that patients with longer CTGn required more nutritional support.^1^ Malnutrition and cardiac health go hand in hand. Malnutrition can lead to muscular atrophy, including weakened cardiac muscle. Nutritional deficiencies can lead to electrolyte imbalances, increase the risk of heart failure, or worsen cardiomyopathies, common conditions associated with myotonic dystrophy.^3^ Also, skeletal muscle atrophy leads to a more sedentary lifestyle that can further pose cardiovascular effects by increasing hypertension and worsening venous return. This could further worsen other heart conditions, such as peripheral vascular disease, leading to sudden cardiac death.^3^

Given the complex nature of DM1, it is crucial to investigate non-cardiac outcomes, such as respiratory insufficiency and malnutrition, and their relationships with cardiac complications to obtain a comprehensive understanding of the causes of sudden cardiac death in the disease. Itoh *et al*.^1^ offer valuable knowledge of worsening cardiovascular outcomes due to the lengthening of CTGn in DM1 patients. However, there is a pressing need for further research on how other non-cardiac events can exacerbate cardiac abnormalities and mortality rates in this cohort of DM1 patients.

## Data Availability

There is no new data associated with this article.
